# Higher Risk for Sensitization to Commonly Consumed Herbs among Adults and Youngsters Suffering from Birch, Mugwort or Grass Pollinosis

**DOI:** 10.3390/ijerph20010033

**Published:** 2022-12-20

**Authors:** Waldemar Wagner, Krzysztof Buczyłko, Aneta Wagner, Angelika Szwed-Kowalska, Anna Stasiak

**Affiliations:** 1 Department of Hormone Biochemistry, Medical University of Łódź, 7/9 Żeligowskiego Str., 90-752 Łódź, Poland; 2 Laboratory of Cellular Immunology, Institute of Medical Biology Polish Academy of Sciences, 93-232 Łódź, Poland; 3 Faculty of Health Sciences, State University of Applied Sciences in Włocławek, 87-800 Włocławek, Poland; 4 Allergology and Respiratory Rehabilitation Department, Medical University of Łódź, 90-752 Łódź, Poland; 5 NZOZ Allergology Center, 90-553 Łódź, Poland

**Keywords:** sensitization to herbs, pollinosis, cross-reactivity, skin prick test, children

## Abstract

Background: According to recent findings, mugwort and birch pollen-allergic patients represent a high-risk group for developing adverse allergic reactions to herbal spices due to cross-reacting allergens found in both pollen and raw herbs. Such associations are known as a pollen-plant food allergy syndrome. Objective: The aim of the study was to evaluate the extent of sensitization to commonly consumed herb species representing Lamiaceae, Apiaceae and Brassicaceae families in Polish patients with suspected birch, mugwort or grass pollen allergy. Materials and Methods: Data were obtained from 180 patients, adults and children with suspected allergy to aeroallergens. Skin prick tests (SPT) were performed with standard birch, mugwort, grass mixture or dust mite extracts. Prick by prick tests were performed with fresh extracts of popular herbs: basil, oregano, lemon balm, mint, salvia, rosemary, thyme, anise, caraway and mustard. Results: Twenty-nine percent of patients were characterized by concomitant positive skin prick reactions to both herbs and pollens extracts. The concomitant pollinosis significantly increased the risk of SPT reaction to all tested herbs in adults (odds ratio, OR = 2.15–7.35) and children (OR = 5.3–28). The extent of SPT responses to herbs from Lamiaceae + Apiaceae were strongly correlated with SPT responses to pollens in the pediatric group (r = 0.685/*p* < 0.001). Conclusion: The study demonstrates that youngsters suffering from pollinosis are at high risk of developing allergic reactions to herbs and highlights the importance of including native skin prick tests with herbs in the diagnostic work-up for suspected food allergy.

## 1. Introduction

Sensitization to herbs commonly used in cuisine and cosmetics, although rare, is being increasingly reported [1]. The prevalence of allergic responses to herbs is growing since people are seeking herbal medicines and cosmetics as an alternative to synthetic ones, as well as these food ingredients being considered to be health-promoting components in the human diet. Clinical symptoms from herbs and their extracts are infrequent but occasionally severe. The adverse reactions to herb constituents may manifest as a contact dermatitis or acute hypersensitivity reactions, such as angioedema, or even life-threatening reactions, such as anaphylactic shock [2,3,4,5,6]. Herbs and their constituents are widely used in the world, in a large variety, often as blends containing multiple allergens, yet spice allergy is undoubtedly underdiagnosed. This is particularly due to the lack of reliable allergy skin test extracts and shortage of available blood tests [7]. Although spice allergy is estimated for 2–4% of food allergies, it is usually misdiagnosed because spice allergens are commonly hidden in many commercial foods [8,9]. 

According to recent findings, common plant pollens and herbs share cross-reactive allergens [10,11,12,13,14]. Thus, patients primarily sensitized to birch, mugwort or grass pollens are prone to developing allergic reactions to herbs due to IgE cross-reactivity to homologues of PR-10 allergens (e.g., Bet v 1 and Api g 1), profilins (e.g., Bet v 2 and Api g 4), nonspecific lipid-transfer proteins (LTPs) or high molecular weight allergens (HMWA, e.g., Api g 5 and Phl p 4) [10,11,12,13,14,15]. For example, patients with mugwort and birch pollen allergy represent a high-risk group for developing food allergies to the herbs belonging to Apiaceae family [15]. Higher rate of sensitization to coriander, caraway, fennel and celery has been reported due to high cross-reactivity of their allergens within the plant family, known as a celery–birch–mugwort–spice syndrome. Recently, allergy to mustard seeds has also been reported [12,13]. The authors have showed significant associations of mugwort pollinosis and Brassicaceae family-derived food allergy, suggesting presence of a new mustard–mugwort allergy syndrome [12,14]. 

Herbs of Lamiaceae family represent the most popular plants and spices used in South Europe and Mediterranean cuisine. This plant family includes aromatic basil, oregano, lemon balm, mint, salvia, rosemary, thyme and others. Since the mid-1990s, there have been limited reports of allergy or allergy-like reactions to basil [3], oregano and salvia [16], oregano and thyme [17], rosemary [2,18,19] and mint [1,4,6]. Nevertheless, the descriptive study of Meincke and Pokladnikova based on extracts of WHO’s VigiBase® highlighted the potential of herbal medicines to cause serious immediate allergic reactions, including those life-threatening adverse drug reactions, such as anaphylactic and anaphylactoid reactions [1,5]. More important, the population of patients under 18 years was reported more prone to suffering from hypersensitivity reactions than the rest of the population.

The aim of the current study was to assess the extent of sensitization to commonly consumed herbs in populations of adults and children, nonallergic and allergic to birch, mugwort or grass pollens. The herbs and spice sensitization is often underrated or misdiagnosed during routine allergy evaluation. The positive skin prick test with herbs may imply patient predisposition to allergic reactions to particular spices in the future. Although, these reactions are rare but may occasionally develop severe manifestations. Thus, an engaged patient may find it beneficial to restrict potential health threatening episodes through control of spices consumption.

## 2. Materials and Methods 

To extend clinical data on sensitization to popular fresh herbs in Central–East Europe, we recruited adult and adolescent patients in Poland with suspected allergy to aeroallergens. Using the skin prick by prick tests, we evaluated the immediate reactions against 10 herbs belonging to Lamiaceae (basil, oregano, lemon balm, mint, salvia, rosemary and thyme), Apiaceae (anise and caraway) and Brassicaceae (mustard) families, in relation to patients’ sensitization status to birch, mugwort or grass pollens. 

### 2.1. Subjects

The study groups included 180 subjects. The mean age of patients in adult (*n* = 117) and pediatric (*n* = 63) groups were 47 and 9 years (4–17 years range), respectively. The study was performed in Allergology Center clinic in Łódź. Patients eligible for participation in the study were interviewed for positive history of birch, mugwort, grass or dust mites allergy, the occurrence of clinical manifestations of allergic rhinitis (118), allergic contact dermatitis (17), bronchial asthma (22), rhinoconjunctivitis (3), atopic dermatitis (2), chronic urticaria (5), cough (4), gastrointestinal allergic disease (3) or skin inflammation (6) during pollen season. During testing procedures patients were supervised by qualified nurses and anesthesiologists. All patients or their legal guardians provided written informed consent prior to participation in the study. The study was approved by the Bioethics Committee of the Regional Medical Chamber in Lodz, No: RNN/731/11. 

### 2.2. SPT and Prick by Prick Test Procedures

Sensitization to pollens and dust mites were confirmed by SPT using standard birch extract, mugwort pollen extracts, grass mixture extract and dust mite extract (Betula alba #108; Artemisia vulgaris #106; grass mixture #006; D. pteronyssinus #708; D. farinae #725; extract concentration 50,000 SBU/mL; Allergopharma, Germany). SPT and prick by prick test with fresh herbs were performed as described previously (20). Briefly, fresh herbs’ leaves (basil, oregano, lemon balm, mint, salvia, rosemary and thyme) were grinded with a mortar and the resulting pulp was used for pricking. Seeds of anise, caraway and mustard were grinded in the presence of an equal volume of 0.9% saline (1:1 *v/v*). All extracts were prepared from herbs and seeds available in the markets. Tests were performed along with histamine dihydrochloride (1.7 mg/mL) as a positive control and SPT diluents (0.9% natrium chloride) as a negative control. The SPT results were regarded as positive when the diameter of the wheal was ≥3 mm. Tests were performed beyond pollen season in Poland (September–February). All participants confirmed that antihistamines were stopped no less than 7 days before testing.

### 2.3. Statistical Analysis

The statistical analyses were performed with the aid of GraphPad 5 software (GraphPad Software, San Diego, CA, USA). The strength and direction of the linear relationship between variables (cumulative SPT results to herbs vs. rank of SPT responses to pollens extracts) were evaluated via Pearson’s correlation coefficient (r). Rank of SPT responses to pollens extracts were assessed on the basis of the number of positive SPT reactions to tested pollen extracts: birch, mugwort or grass (0—negative SPTs, 1—one positive SPT, 2—two positive SPTs, 3—three positive SPTs).

## 3. Results

**Figure 1:**
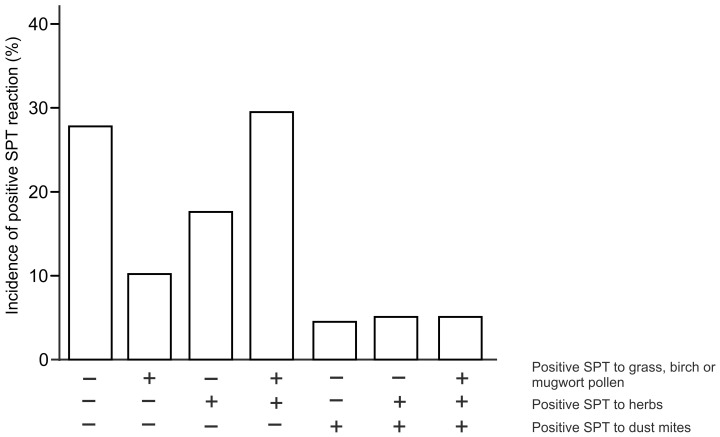
Incidence of single or concomitant positive SPT reactions to standardized birch, mugwort and grass pollen extracts, standardized dust mite extracts and fresh herbs extracts in the studied group of patients (*n* = 180), adults and children.

We studied the immediate allergic reactions to commonly consumed herbs using SPTs in respect to their allergy status to birch, mugwort, grass and dust mites. In the studied group of subjects (*n* = 180), 10% of patients were diagnosed as SPT-positive to birch, mugwort or grass pollens, 18% of patients were diagnosed as SPT-positive to the studied 10 herb species and 4.5% of patients were diagnosed as SPT-positive to dust mites. Almost 28% of enrolled subjects were SPT-negative to tested allergens (Figure 1). Interestingly, a detailed examination of patients allergic to pollen allergens and herb extracts revealed that 30% of patients developed concomitant positive allergic skin reaction to herb and pollen extracts. Consequently, concomitant allergic response for herbs and dust mite extracts was observed only in 5% of patients. Indeed, dust mites and plants do not share cross-reactive allergens due to their phylogenetic distinctiveness and concurrent skin reactions to these extracts being rather unlikely to observe. 

**Figure 2:**
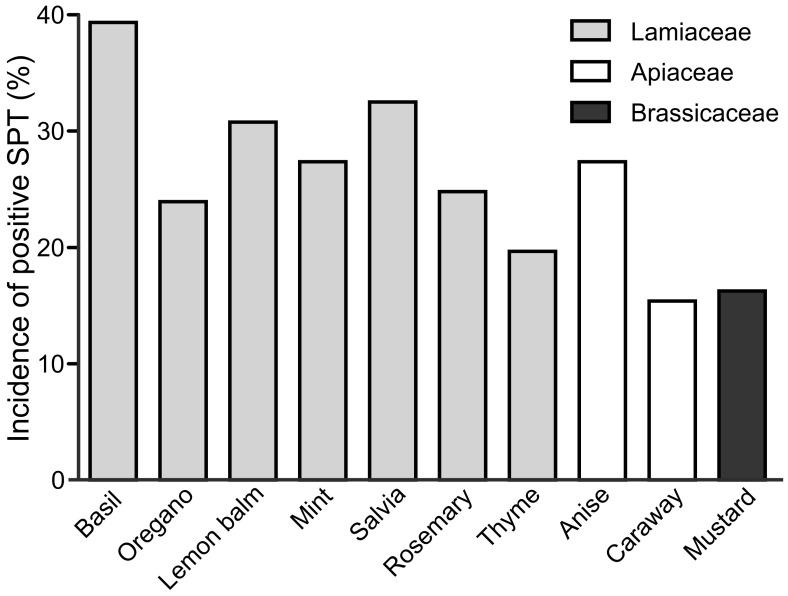
Incidence of positive SPT reaction to fresh extracts of basil, oregano, lemon balm, mint, salvia, rosemary, thyme anise, caraway and mustard in a group of adults (*n* = 117).

**Figure 3:**
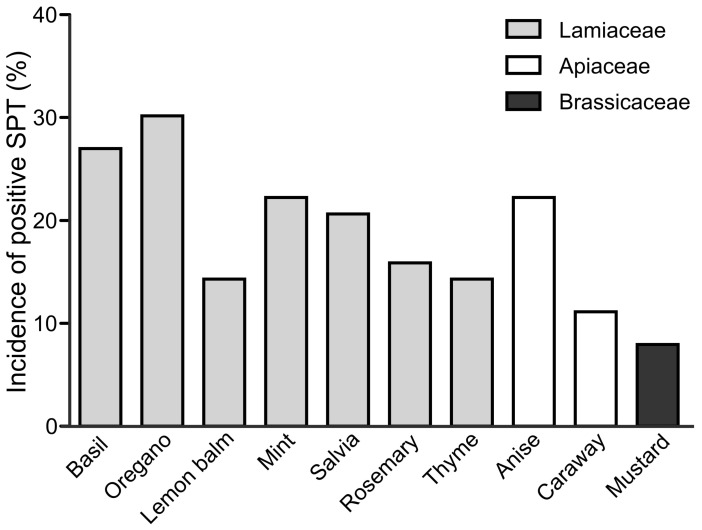
Incidence of positive SPT reaction to fresh extracts of basil, oregano, lemon balm, mint, salvia, rosemary, thyme anise, caraway and mustard in pediatric group (*n* = 63).

Further, we evaluated the susceptibility of the adult and pediatric patients to developing SPT reactions to particular herb species. We tested 10 herb species commonly used in cuisine: basil, oregano, lemon balm, mint salvia, rosemary, thyme, anise, caraway and mustard belonging to three different plant families: Lamiaceae, Apiaceae and Brassicaceae (Figure 2 and Figure 3). Regardless of the pollinosis status of adult patients, the incidence of SPT reactions to Lamiaceae herbs was as high as 39% for basil, 24% for oregano, 31% for lemon balm, 27% for mint, 32% for salvia (32%) and 25% for rosemary (Figure 2). Apiaceae herbs were responsible for 27% and 15% of positive SPTs for anise and caraway, respectively, while skin prick with mustard seed extract evoked skin reaction in 16% of adult patients.

Pediatric patients showed similar distribution of SPT reactions to herbs, although overall incidence of positive SPT responses was markedly lower, except for oregano. The highest incidence of SPT reactions to herbs among children were found for basil, oregano, mint, anise and salvia (27%, 30%, 22%, 22% and 21%, respectively; Figure 3). It is worth noting the higher incidence of positive SPT reaction to oregano in children than in adult patients. Caraway and mustard were the least SPT-reactive (11% and 8% accordingly).

According to the study by Jensen-Jarolim and colleagues, the allergens homologous to birch pollen Bet v 1 and Bet v 2 may be responsible for Type I allergy to herbs of Apiaceae family [15]. To further explore this hypothesis, we investigated the effect of sensitization to pollens (birch, mugwort or grass) on the incidence of positive SPT reaction to particular herbs species in adult and pediatric groups of patients. The analysis showed that concomitant pollinosis significantly increases the risk of allergic reaction to all tested herbs in adults (OR = 2.15–7.35) (Table 1). The highest risks of positive SPT were found for the herbs belonging to Lamiaceae and Apiaceae families. These adult patients with pollinosis responded 5.4, 7.3 and 5.4 times more often to thyme, anise and caraway, respectively, than their corresponding counterparts without pollinosis. Surprisingly, although the similar pattern of responses to SPT with herb extracts was found in children, the risks of positive response of pediatric patients with pollinosis exceeded even twice that observed for adults (except for oregano) (Table 2). Pediatric patients with pollinosis responded 16–18 times more often to herbs of Apiaceae family, while even 28 times more often to lemon balm (Lamiaceae family) than children not suffering from pollinosis. In other words, the concomitant sensitization to pollen significantly increased the chance (odds ratio, OR) of positive SPT reaction to herbs.

Furthermore, pediatric patients with history of pollinosis, confirmed by SPT, exhibited the highest chance of positive response to herbs, which exceeded two- to four-fold OR observed in adults. Subsequently, we drew plots showing the association between rank of SPTs for pollens (0, 1, 2 or 3) and SPT results for herbs and calculated the corresponding Pearson’s correlation coefficients. As shown in Table 3, the extent of positive responses to herbs from Lamiaceae and Apiaceae were unequivocally correlated with rank of SPT for pollens in both adult and pediatric groups, and these correlations were distinctly higher in the pediatric group of patients. It is worth emphasizing the almost two-fold higher degree of concomitant sensitization to Apiaceae herbs and birch, grass or mugwort pollens among children in comparison to adults (r = 0.767 vs. r = 0.433).

As expected, neither such association of concomitant dust mites and sensitization to herbs (Figure 1) nor higher incidence of positive SPT to herbs in patients allergic to dust mites (Table 4) was observed.

**Figure 4:**
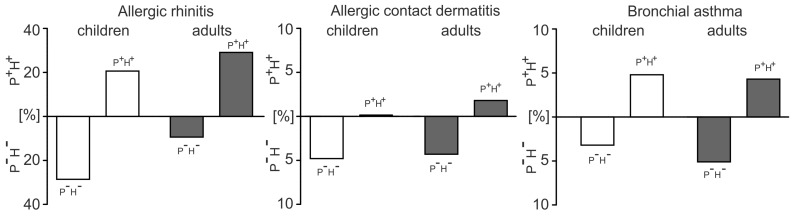
The incidence of concomitant sensitization to pollens and herbs (P^+^H^+^) or lack of such association (P^−^H^−^) in adults (*n* = 117) and children (*n* = 63) suffering allergic rhinitis, allergic contact dermatitis or bronchial asthma.

In the next step, we performed more detailed analysis within adults and children subgroups to evaluate the association of concomitant sensitization to birch, mugwort or grass and herbs (Lamiaceae + Apiaceae families) in regard to allergic diseases of patients. Allergic rhinitis, allergic contact dermatitis and bronchial asthma were analyzed, as they were the most represented in the study group (Figure 4). We compared distribution of patients concomitantly sensitized to pollens and herbs (P^+^H^+^) against nonsensitized ones (P^−^H^−^). 

The obtained results confirmed strong association of pollens and herbs sensitization in children suffering from allergic rhinitis as compared to adults. “Naïve” pediatric patients in respect to pollen and herb sensitization (P^−^H^−^) prevailed over the respective group P^−^H^−^ of the adult patients (29% vs. 9%). However, following concomitant sensitization to pollens and herbs, the incidence of P^+^ H^+^ patients among children and adults reached the similar extent (21% and 29%, respectively). For bronchial asthma, the incidence of P^+^H^+^ were at a similar level (5% vs. 4%), while patients suffering from allergic contact dermatitis barely accounted for concomitant sensitization P^+^H^+^.

## 4. Discussion

Allergies to plant foods often develop due to allergic sensitization to respiratory allergens. Such associations are known as a pollen-plant food allergy syndrome. For example, allergy to apples is the consequence of Bet v 1 birch pollen allergen sensitization and subsequent IgE and T-cell cross-reactivity with homologous allergens, such as Mal d 1 in apple [20,21]. In central and northern Europe, birch pollen is responsible for almost 20% of overall pollen allergy and accounts for birch–plant food syndrome symptoms in 70% of the patients with pollinosis [22]. In the celery–mugwort–birch–spice syndrome, IgE cross-reactivity is associated with several classes of allergens: PR-10 (Bet v 1 and Api g 1), profilins, such as Bet v 2 and Api g 4, nonspecific LTPs and high molecular weight allergens (HMW), such as Api g 5 and its homologues from grass pollen and fennel [10,23]. Other pollen-plant associations reported in the scientific literature include mugwort–mustard and other Brassicaceae family plants syndrome, Ambrosia–melon–banana syndrome, Parietaria–pistachio, olive–plant foods syndrome and others [12,14,15,23]. According to recent findings, mugwort and birch pollen allergic patients represent a high-risk group for developing plant food allergy not only to celeriac, but also to closely related herbs from Apiaceae family (anise, fennel, coriander and cumin) due to constituents of homologue proteins to Bet v1, Bet v 2 and 60 kD HMW allergens [15]. Although, herbs of Lamiaceae family, such as aromatic basil, oregano, lemon balm, mint, salvia, rosemary and thyme, represent the most popular plants and spices used in the South European and Mediterranean cuisine, there are limited reports indicating allergy to these spices. There have been isolated reports of allergic reactions to mint [1,4,5,6], salvia [15], oregano [15,16] or skin irritation (contact dermatitis) to rosemary [2,18,19]. Since popular spices from Lamiaceae, Apiaceae and Brassicaceae share cross-reactive allergens and are commonly used in cuisine, it is suspected that their role in development of allergic reactions might be underestimated. 

To our knowledge, there is a lack of extensive study evaluating the allergenic potential of spices in large experimental groups based on SPT testing with native herbal extracts. In this work, we evaluated the allergic reaction to commonly consumed herbs in the adults and children populations in Central–East Europe sensitized to birch, mugwort or grass pollens. We examined the reactions of 180 patients to 10 herbs’ extracts using SPTs and assessed the involvement of IgE-dependent reactions to these plants’ challenge. SPT testing with fresh food that contains native allergens is widely accepted as a clinical tool for the assessment of sensitization to a particular food [13]. Within the examined cohort, one third of patients were plant-pollen-sensitized and concomitantly prone to positive SPT response to herb extracts (30%). Contrarily, patients positively responding only to single SPTs with birch, mugwort and grass extracts or to SPTs with herb extracts only accounted merely for 10% and 18% of tested subjects, respectively. These results are in line with the results of the previous meta-analysis study, emphasizing that patients at risk of spice allergy are young adults sensitized to mugwort and birch allergens, sharing cross-sensitization with various food vegetal allergens [23]. Indeed, as shown in Figure 1, there were two to three times more patients concomitantly sensitized to pollens and herbs than to pollens or herbs alone. As expected, neither such association of concomitant dust mites and herbs allergy (Figure 1; Table 4) nor higher incidence of positive SPT to herbs in patients sensitized to dust mites was observed (thus, dust mites tests could serve as a negative control). Analysis of incidence of positive SPT to particular herbs showed that adults responded positively to tested herbs at rates of 15% (mustard)−39% (basil), while pediatric patients exhibited lower incidence of positive SPT to herbs, accordingly, at rates 8% (mustard)−30% (oregano). As suspected, shorter time of exposition to pollens of youngsters translated into a lesser degree of sensitization to aero-allergens through their lives and lower responsiveness to herbs’ extracts (Figure 3). Indeed, calculation of the risk of positive SPT for particular herbs in respect to the presence or absence of pollinosis indicated clear association of allergic response to herbs and concomitant pollinosis in children and adults. More importantly, the observed risk of allergy development to herbs was more evident (OR = 5.3–28) in children in comparison to adults (OR = 2.1–7.35), emphasizing the major role of pollen sensitization over other cross-reactive sensitizing allergens in the human lifespan (Table 1 and Table 2). 

Finally, we demonstrated strong correlations between SPT results for herbs and birch, grass or mugwort pollinosis status in pediatric and adult patients. These results indicated strong relationship between extent of pollen sensitization (1–3) and allergic reactions to herbs, notably for plants from Apiaceae family (r = 0.767, *p* < 0.001) in pediatric patients (Table 3). Collectively, by plotting pollinosis ranks and SPT results for herbs, we could clearly show the role of timespan and components of sensitizing allergens in the strength of allergic response and differentiate allergic responsiveness to herbs between children and adults. Furthermore, these results were supported by subsequent analysis of distribution of patients concomitantly sensitized to pollens and herbs (P^+^H^+^) or not (P^−^H^−^) in particular allergic subgroups of patients. Children suffering from allergic rhinitis were predominantly represented in the P^−^H^−^ group, as compared to adult patients; however, once sensitized, children accounted for the P^+^H^+^pattern at the comparable level to adults (Figure 4). A similar pattern was observed in bronchial asthma patients and not the allergic contact dermatitis group but, due to the relatively small size of these particular experimental groups, the results obtained should be interpreted with care. Since we could observe more evident translation from P^−^H^−^ pattern to P^+^H^+^ in children, we may tentatively assumed higher susceptibility of pediatric patients to pollen-related sensitization to tested herbs. Collectively, our study, with use of a multifaceted analysis approach, provides the evidence of higher risk for sensitization to commonly consumed herbs among adults and youngsters suffering from birch, mugwort or grass pollinosis. Importantly, the similar conclusion was drawn from the previous study on adverse drug reactions toward herbal medicines in children. It has been shown that children at age 7–12 are at higher risk of suffering from allergic-like reactions [5]. 

However, this study may have a few limitations. First, the experimental groups of adults and children were not equal, and the adult group was almost twice the pediatric one. Moreover, the groups were not homogenous in regard to reported or suspected allergic diseases, although allergic rhinitis, allergic contact dermatitis and bronchial asthma (total 157 cases) were the predominant allergic diseases diagnosed in both groups. Another potential confounding bias might be introduced by some patients controlling their bronchial asthma by use of corticosteroids or use nonsteroidal anti-inflammatory drugs against fever in children, which was not reported to investigators. 

## 5. Conclusions

In summary, this study provides clinical evidence of sensitization to herbs commonly used in cuisine. Our survey demonstrated 53.3% incidence of immediate SPT responses to basil, oregano, lemon balm, mint, salvia, rosemary, thyme, anise, caraway and mustard in Polish patients. Among this group, 33.9% of patients were concomitantly sensitized to birch, mugwort or grass pollens, thus indicating the clinical relevance of this outcome in food allergy diagnostics. Furthermore, children suffering from allergic rhinitis were prone to developing concomitant sensitization to herbs and birch, mugwort or grass pollens. Particularly, the demonstration that children suffering from pollinosis exhibit higher risk of SPT reactions to herbs than adults highlights the importance of including native tests with herbs for suspected food allergy in early teens. The herbs and spice sensitizations are often underrated or misdiagnosed during routine allergy evaluation. Importantly, positive skin prick test with herbs may be indicatory for a patient to restrain potential health-threatening episodes through control of spice consumption.

## Figures and Tables

**Table 1:** Incidence and odds ratio (OR) of positive SPT (Φ ≥ 3 mm) for particular herbs in relation to positive (+) or negative (−) SPT for pollen extracts in adults.

Herb Species	SPT for Pollen Extracts (+)		SPT for Pollen Extracts (−)		OR	95% CI	*p* Value
	*n*	%	*n*	%			
Basil	31	26.5	15	12.8	2.519	1.162–5.461	0.023
Oregano	22	18.8	6	5.1	4.293	1.588–11.60	0.004
Lemon balm	24	20.5	12	10.2	2.154	0.949–4.885	0.073
Mint	24	20.5	8	6.8	3.538	1.429–8.764	0.006
Salvia	27	23.1	11	9.4	2.932	1.279–6.719	0.011
Rosemary	22	18.8	7	6	3.603	1.396–9.3	0.009
Thyme	19	16.2	4	3.4	5.398	1.706–17.08	0.002
Anise	27	23.1	5	4.3	7.35	2.580–20.94	<0.001
Caraway	15	12.8	3	2.6	5.426	1.476–19.94	0.009
Mustard	15	12.8	4	3.4	3.906	1.210–12.61	0.023

Abbreviations: SPT, skin prick test; OR, odds ratio; 95% Cl, 95% confidence interval.

**Table 2:** Incidence and odds ratio (OR) of positive SPT (Φ ≥ 3 mm) for particular herbs in relation to positive (+) or negative (−) SPT for pollen extracts in children.

Herb Species	SPT for Pollen Extracts (+)		SPT for Pollen Extracts (−)		OR	95% CI	*p* Value
	*n*	%	*n*	%			
Basil	11	17.5	6	9.5	7.537	2.196–25.87	0.002
Oregano	11	17.5	8	12.7	5.347	1.661–17.21	0.007
Lemon balm	8	12.7	1	1.6	28	3.177–246.7	<0.001
Mint	9	14.3	5	7.9	6.218	1.724–22.43	0.007
Salvia	9	14.3	4	6.3	7.977	2.058–30.92	0.002
Rosemary	7	11.1	3	4.8	7.179	1.617–31.88	0.008
Thyme	7	11.1	2	3.2	11.04	2.034–59.89	0.003
Anise	11	17.5	3	4.8	16.30	3.756–70.70	<0.001
Caraway	6	9.5	1	1.6	18	1.990–162.8	0.003
Mustard	4	6.3	1	1.6	10.5	1.089–101.3	0.032

Abbreviations: SPT, skin prick test; OR, odds ratio; 95% Cl, 95% confidence interval.

**Table 3:** Correlations between SPT results for herbs and SPT results for birch, grass or mugwort pollen extracts in pediatric and adult patients (r/*p*-value).

	Cumulative Positive SPTs for Herbs	Lamiaceae + Apiaceae	Lamiaceae	Apiaceae
Rank of SPT for Pollens				
Pediatric patients		0.685/<0.001 95% CI 0.529–0.796	0.594/<0.001 95% CI 0.409–0.732	0.767/<0.001 95% CI 0.643–0.851
Adult patients		0.451/<0.001 95% CI 0.293–0.586	0.415/<0.001 95% CI 0.252–0.556	0.433/<0.001 95% CI 0.273–0.571

Abbreviations: SPT, skin prick test; r, Pearson’s correlation coefficient; 95% Cl, 95% confidence interval.

**Table 4:** Incidence and odds ratio (OR) of positive SPT (Φ ≥ 3 mm) for particular herbs in relation to positive (+) or negative (−) SPT for mite extracts.

Herb Species	SPT for Mites Extracts (+)		SPT for Mites Extracts (−)		OR	95% CI	*p* Value
	*n*	%	*n*	%			
Basil	12	6.7	51	28.3	2.059	0.8647–4.902	>0.05
Oregano	7	3.9	40	22.2	1.194	0.4614–3.091	>0.05
Lemon balm	8	4.4	37	20.5	1608	0.6373–4.058	>0.05
Mint	9	5	37	20.5	1.930	0.7805–4.771	>0.05
Salvia	10	5.5	41	22.8	2.003	0.8256–4.862	>0.05
Rosemary	7	3.9	32	17.8	1.596	0.6095–4.177	>0.05
Thyme	7	3.9	25	13.9	2.158	0.8107–5.743	>0.05
Anise	7	3.9	39	21.7	1.235	0.4767–3.201	>0.05
Caraway	4	2.2	21	11.7	1.276	0.3968–4.105	>0.05
Mustard	4	2.2	20	11.1	1.360	0.4213–4.390	>0.05

Abbreviations: SPT, skin prick test; OR, odds ratio; 95% Cl, 95% confidence interval.

## Data Availability

Not applicable.
